# Troublesome Bodies: How Bodies Come to Matter and Intrude in Eating Disorder Recovery

**DOI:** 10.1007/s11013-025-09896-6

**Published:** 2025-02-09

**Authors:** Mari Holen, Agnes Ringer, Anne Mia Steno

**Affiliations:** 1 Department of People and Technology, Roskilde University, Universitetsvej 1Building 44.1., Postbox 260, 4000 Roskilde, Denmark; 2 University College Copenhagen, Copenhagen, Denmark

**Keywords:** Eating disorders, Recovery, Bodies, Space

## Abstract

Understanding how bodies come to matter in eating disorder recovery is complex, particularly given the unresolved question of whether eating disorders are fundamentally about the body. Drawing on Analu Verbin’s adaptation of Judith Butler’s theory of performativity and Sarah Ahmed’s body phenomenology, this paper examines how participants in a narrative and systemic group therapy program at a mental health clinic for eating disorders perceive themselves as recovering or recovered. We explore how the body is presented and understood in their recovery narratives, developing the concept of the ‘troublesome body’ to highlight the ambiguities these narratives reveal. The body in the participants’ narratives is continuously shaped by an external gaze that alternates between recognition and concern, often oscillating between praise and scrutiny. Participants are tasked with cultivating a liberated, sensual body and a more natural relationship with food, achieved through therapeutic strategies such as establishing a mechanical eating pattern and ‘neutralizing’ the body in group settings. Yet the body resists, asserting its presence through physical sensations—rumbling stomachs and ‘blobby’ forms—that challenge these efforts. Crucially, the narrative and systemic group therapy is viewed by participants as pivotal in their recovery not because it resolves all eating disorder-related issues, but because it offers a collective space for ‘troublesome bodies.’ This space allows for bodies to exist without conforming to societal dichotomies or norms that are often imposed in other treatment contexts, thus, offering an alternative model of recovery where bodily ambiguities can be embraced rather than resolved.

## Introduction

Eating disorders (EDs) are contradictory and paradoxical conditions, categorized as mental disorders but manifesting primarily on the physical body. The unclear role of the body is reflected in the way the body is approached in the treatment of EDs and the way people with EDs are expected to recover. Much clinical research and practice have approached the eating disordered body as quantifiable biological data, focusing on factors such as BMI, the degree of restrictive eating, or the number of compensatory behaviors. Discussions on the relationship between identity and the body are in the clinical literature often confined to notions of ‘body image disturbances’, focusing on the individual and the mind (Gabrielli & Irtelli, 2022). This strand of research, however, overlooks that experience is embodied and that the mind and body are always situated in a social and cultural context. Sociological perspectives emphasize that eating disorders reflect cultural and social norms related to gender (Hepworth, 1994; Bordo, 2003). While the sociological literature is important, there is arguably a tendency in some social theory accounts to view anorexic bodies as ‘empty vessels’ (Eli & Lavis, 2021) molded by ‘the stamp of prevailing historical forms of selfhood, desire, masculinity, femininity’ (Bordo, 2003). Such an understanding may not fully convey the embodied experience of living with an eating disorders and may overlook that eating disordered behavior is agentic in its own right.

In recent years, there has been a renewed interest in the embodied experiences and the bodily aspects of eating disorders among feminist researchers (Eli, 2016; Eli & Lavis, 2021; LaMarre & Rice, 2016). These perspectives recognize that bodies are not just ‘acted upon’ by external or internal influences (LaMarre & Rice, 2016). For example, Rebecca Lester (2019) has argued that far from being disembodied or passive, people with eating disorders work actively and deliberately through their ED behavior to navigate the conditions of their existence in a specific social context. Eating disordered practices read as pathological and problematic in a treatment framework may be empowering to the person engaging in them (if at least temporarily). This helps explain why recovery from eating disorders may be ambivalent.

Medical anthropologists Karin Eli and Anna Lavis (2021) have pointed out the need for increased attention to how eating disorders live and function in the spaces of people’s everyday life, and how relationships and interactions among people, artifacts, and places affect, sustain, and resist eating disorder practices. They illustrate that eating disorders are deeply connected to the contexts and environments they exist in. Contexts should not be understood (solely) as cultural images and texts that produce idealized images, but as concrete things we surround ourselves with and specific spaces that discipline and ‘whisper’ stories about how bodies are constructed and seen.

Nevertheless, debates surrounding the embodiment of eating disorders and the fundamental question of whether they are about the body at all remain unresolved. In particular, the role of the body in the process of recovery from eating disorders has been scarcely researched. In this article, we build on Eli and Lavis (2021) suggestion that although eating disorders are enacted on the body, they are not essentially about the body; they are embodied but not bodily. Drawing on Lester (2019), Eli & Lavis argue that rather than representing facts of the body, ‘eating disorders represent dynamic flows that speak to cultural concerns with boundaries and relatedness’ (Lester, 2019; cited in Eli & Lavis, 2021). On a similar note, LaMarre & Rice have argued that the body in eating disorders should be understood as intervowen (LaMarre & Rice, 2016, 2021; Rinaldi et al., 2016). By this, they mean that the eating disordered body acts agentively in a cultural space, intimately connected to other bodies through strings of relationships, which may be conflictual and ambivalent.

Given the above conception of eating disorders as culturally embodied—and the body as an agentive actor—there is a need to explore the role of the body in eating disorder recovery. Individuals recovering from eating disorders live in a world, where obesity is considered unhealthy and prescriptions for ‘healthy eating’ are omnipresent. They live in a world where the actions of monitoring food intake, exercising and losing weight are culturally valued, as they reiterate recognizable social codes, particularly of femininity (Butler, 1993). In such a context, the ostensibly simple question of what recovery entails and at which point a person may be considered ‘recovered,’ becomes elusive and ambiguous. The complexities surrounding the body in EDs and the cultural and interpersonal space, thus, become particularly pronounced as people struggle to recover from eating disorders. In this paper, we draw on interviews with participants who consider themselves recovering or recovered from EDs to explore how the body is present(ed) in their narratives of recovery.

The participants of the study have all participated in a narrative and systemic group therapy program in Denmark. The purpose of the treatment was to create a language for the emotions, experiences, and ways of coping that eating disorders are entangled in and to develop new ways of relating to them (Albinus & Østergaard, 2021). This therapy approach represents in many ways a departure from traditional treatments of eating disorders, which often place significant emphasis on body markers, such as weight management and prescribing behavioral change. We found it interesting to explore how participants talked about and related to their bodies after having completed a therapy program, which did not have the body as a primary focus in the treatment of eating disorders.

## Theoretical Perspective

The paper is theoretically informed by Analu Verbin’s(2020) work, which is based on Judith Butler’s performativity and queer theory (Butler, 1990, 1993). In discussing anorexia, Verbin(2020) has argued that the essence of the condition is paradox and contradiction. Verbin delineates three fundamental paradoxes that she argues point to the body’s role as both the battleground and weapon of feminine conflicts. The first paradox revolves around femininity/non-femininity; in the pursuit of the ‘ideal’ slim feminine physique, any physical manifestations of femininity, including menstruation, are suppressed, or rejected. The second paradox involves control/lack of control; anorexia is at once the ultimate control of internal desires (hunger, appetite, needs) and a complete loss of control (an inability to cease with anorexic actions, even when attempting to do so). The third paradox concerns the relationship between the body and mind. Anorexia is portrayed as the triumph of mental willpower over bodily needs, yet anorexics also express the opposing notion that the mind, or emotions, are feeble and evil, and the only reliable entity is the body.

We combine this perspective with Sarah Ahmed’s bodily phenomenology, which emphasizes that while bodies are mutable and the mind is central (as eating disorders demonstrate), the body cannot be made to disappear. Bodies are never neutral; they matter and take shape through interactions with others. At times, they are loud and commanding; at other times, they fade into the background or create hierarchies. Yet, they are always present, an inescapable reality of being in the world (Ahmed, 2006).

## Methodology

This study employed a qualitative design, using in-depth semi-structured interviews with patients and ‘co-interpretative workshops’ (Lincoln & Guba, 1985) during which professionals and former patients were invited to reflect on excerpts from the patient interviews. This allowed for a breadth in perspectives, and for new perspectives to emerge. The original study’s aim was to explore social and collective processes in recovery from eating disorders (EDs). While the original aim of the study was to understand how collective therapeutic and everyday practices contribute to recovery, a prominent theme that emerged during the interviews and workshops was the body—particularly how it functioned as a point of intersection between the individual and the surrounding world. This theme became the focus of the analysis for this paper.

### Study Setting

The study took place at a center specializing in eating disorders, where the treatment program was structured around group therapy sessions inspired by narrative, systemic, and response-based practices (Rasmussen, 2010, 2015; Wade, 1997; White, 2007). These therapeutic approaches view EDs as a means of coping with adverse life experiences or everyday struggles (Albinus & Østergaard, 2021). Group therapy sessions centered on exploring narratives, relationships, and social contexts to collaboratively develop new stories beyond the eating disorder. The treatment lasted 20 weeks with a three and a half-hour group therapy session once weekly. Each group consisted of two therapists and seven patients. The group therapy sessions were structured on individual interviews with each patient and reflections from the rest of the group and one of the therapists, in accordance with the ideas of a reflective team (Andersen, 1991; Rasmussen, 2010).

Participants also attended individual sessions with a dietitian, where the focus was on managing eating habits and exercise routines. The program had ‘non-negotiables’ tied to weight gain or weight stabilization based on the participants’ BMI. Some participants also took part in a ‘contemplation group’ prior to the main treatment, based on the principles of Motivational Enhancement Therapy (Schousboe & Rasmussen, 2012).

### Interviews

We conducted in-depth semi-structured interviews with five participants who had completed the ED program and considered themselves significantly improved. The interviews were structured around our original research aim—exploring the role of collective processes in recovery, especially in the context of group-based therapy. Participants were asked to describe their process of recovery and what had helped themfiltered through the gazes of others. We also asked specifically how their process of recovery was influenced by group dynamics and collective interactions in the group, as well as interactions with friends, colleagues, family, and other people of significance. The interviews lasted 1–2 h and were all conducted by the first author.

A prominent theme that emerged during these interviews was the role of the body in recovery. Participants frequently discussed the body as a site of tension, where personal experiences intersected with societal and environmental influences. As this theme was pervasive and deeply intertwined with the collective aspects of recovery, we chose to focus our analysis in this paper on the body as a key concept in the recovery process.

### Workshops

We organized two interpretation workshops to engage participants in co-interpretation of the data:

#### Professional Workshop

Ten professionals from the clinic participated, including social workers, nurses, physiotherapists, psychologists, and dietitians. Apart from two dietitians, all professionals were trained psychotherapists and had experience as group therapists. The workshop involved reading anonymized excerpts from the patient interviews and discussing their interpretations in relation to their professional practice. The two-hour session was recorded and transcribed for analysis.

#### Former Patient Workshop

Two former patients who had completed the group therapy program more than a year prior to the interview and self-identified as recovered participated. The choice to involve former patients in co-interpretation of the interviews was based on a wish  to capture different perspectives and personal experiences of recovery from different points in time. The workshop involved reading anonymized excerpts from the patient interviews and discussing the former patients’ interpretations in relation to their own personal experiences. The small number of participants allowed for in-depth reflection. Participants offered their interpretations of the interview excerpts and shared how the themes resonated with their own recovery experiences.

Both workshops were designed to elicit further insights into how recovery, collective processes, and the body were perceived by both professionals and former patients.

### Recruitment

The participants for the patient interviews were recruited by the professionals at the treatment center. The inclusion criteria for the selection of participants for the patient interviews were that participants were toward the end of their treatment and perceiving themselves as significantly improved. To maintain reflexivity and avoid potential conflicts of interest, clients of one of the researchers, who also worked as a therapist in the clinic, were excluded from the study. All participants provided informed consent, and the study was approved by the Danish Capital Region’s internal data department, adhering to ethical guidelines.

Participants for the professional workshop were recruited from the clinic staff, and all professionals were invited to participate. The former patients in the second workshop were recruited via a snowball method, with the inclusion criterion being their identification as recovered from their eating disorder and having finished treatment at least a year prior to the study. In addition, they were selected based on having a special and broad interest in the field of eating disorders to ensure that their perspectives would contribute to a deep, critical analysis.

### Analytical Strategy

During the analysis, we focused on how notions of the body appeared in different ways across the interviews and workshops. Initially, the body was relatively absent in the professionals’ discourse and the written materials describing the program. However, in other data—particularly in participants’ personal narratives—the body emerged as a crucial and complex theme.

The participants frequently resisted framing the body as the core issue of eating disorders in a purely medical sense. Instead, they discussed the body in nuanced ways, highlighting tensions between personal experiences of the body and societal expectations. In addition, traces of the body emerged in interviews through physical experiences, such as audible stomach rumblings, and in stories about bodily discomfort.

Our initial thematic coding (Braun & Clarke, 2006) of the interview data focused on these varying representations of the body, leading to the development of the concept of ‘troublesome bodies’. The concept encapsulates the idea that the body is not a passive or neutral entity, but often becomes a site of conflict or tension for individuals recovering from eating disorders. The concept highlights the complexities of embodiment in the recovery process, as the data indicated that the body becomes both ‘troubling’ and ‘troubled’ in several ways. In the next step of the analytical process, we outlined analytical questions that guided our subsequent analytical process:

- Which external influences ‘trouble’ the body and impact on how participants perceive and relate to their bodies?
- How does the body become an active participant in the process of recovery? I.e., how does the body potentially ‘trouble’ its surroundings and the participants’ attempts at recovering from ED?
- Which potential contradictions or paradoxes emerge in participants’ narratives about the body, and how do these reflect broader challenges in the recovery process?

We analyzed the data asking these questions, and the process resulted in three analytical themes, which will be elaborated in the analysis:

- Bodily Adjustments and Troubling Recognition

This theme examines how the external gaze shapes both the development of, and recovery from, an eating disorder. A key issue that emerged in the interviews was the desire for recognition—gaining visibility in the eyes of others. This theme delves into the paradox of simultaneously wanting and resisting being seen and acknowledged as recovering from an eating disorder.

- Untroubling the Body

This theme explores how participants experienced their bodies in different spaces during recovery, with a particular focus on the space of group therapy. Although participants initially feared that their bodies would stand out and be scrutinized within the group, they came to see it as the one space where their bodies ceased to be significant or troublesome. This unexpected shift, described by many as paradoxical, highlights the group’s unique role in easing bodily preoccupation.

- Navigating a Mechanized Sensual Body

This theme addresses the participants’ challenges in relating to their bodies as both mechanistic and sensual. In treatment, they are required to adhere to structured eating patterns and approach recovery as a task demanding effort and discipline. Yet, they simultaneously strive to reconnect with their bodies as living, sensual organisms. This theme explores the tension between viewing the body as a mechanistic entity and as a source of sensual experience.

## Analysis

### Bodily Adjustments and Troubling Recognition

The first theme of the analysis concerns the question of recognition and visibility in the gaze of others prior to treatment. The participants talk in different ways about how disciplining their body has been a common thread throughout their lives, also prior to it becoming a problem for them. As Lea explains:

L: ‘All the time when I went to school, [it] was all about losing weight, and it was so positive (mm) when I lost weight, because it was a goal I had set. Um, and [it] was recognized when I sort of succeeded, by many people and [in many] places […] It’s something you discover how to do (mmm), how to control your food intake, and to lose weight (mmm). And when you lose weight, you gain recognition and praise from the outside world.’

I: ‘Who did you receive praise and recognition from?’

L: ‘Well, both my close relations, but also, uh, working relations, uhm, even from people, like, further away too.’

Lea describes how losing weight and the bodywork involved in the process gave her access to appreciation. Her body and her persona gained visibility and became recognized as successful by others. In a later extract, she similarly describes losing weight as an arena where she ‘could make it’, in contrast to her schoolwork, where she did not achieve the results she hoped for, despite working hard. For Lea, as for many other participants, however, there came a point when her bodywork, previously admired as indicative of willpower, suddenly became perceived in a different light:

‘It all came to a point where people started to worry about me. And they told me not to lose any more weight. They asked if I was okay, asked my loved ones, through my boyfriend and through my parents, erm, so it became, at the time it all sort of went from recognition to becoming a concern.’

Verbin (2020) discusses how the common practices of dieting, food monitoring and exercising constitute repetitive practices and citations of doing ‘real woman’ (i.e., a slender woman). Taking these practices to the extreme produces a distorted version of the traditional feminine model, a kind of parodic mis-citation of femininity. The shift in social responses that Lea encountered thus mark a point when her bodywork could no longer be recognized as successful femininity. Instead, her body is met with a gaze of concern. Lea explains how this created a shift in her own perception: ‘I think I had lost too much weight. I was too thin, I didn’t feel good in my body, so I didn’t like looking at myself because I was so thin’.

The excerpt demonstrates how Lea’s perception of her body is intervowen with other bodies and other’s perceptions. When Lea looks at herself in the mirror, she is not the only one looking on. When others around her worry, she notices how her body is ‘too thin’. Her gaze is filtered through the gazes of others, making her body stand out—or visible—as separate and troubling to others. Her body thus transitions from invisible (unremarkable) to suddenly becoming visible (being noticed and commented upon as a successful, ‘real’ woman’s body)—to suddenly becoming too visible, standing out in an troubling way. Lea describes how this ‘overvisibility’ created an ambivalent position for her:

‘But at the same time, I could imagine that deep down, it still provided a sense of recognition, because it has long been a goal to be thin (mmm) even though I didn’t feel good about it (mm) there was still, in some way, a confirmation that people could see that I had lost weight.’

Lea points to a core ambivalence in eating disordered bodywork; she simultaneously wishes for her body to be seen and not to be seen by others. She feels uncomfortable about her body, yet she wishes for it to bear witness to her accomplishment of weight loss and being in control. At the same time, her body’s troubling ‘overvisibility’ and Leas unsuccessful attempts at weight gain imply a lack of control, as she feels that she no longer can decide for herself (Verbin, 2020).

Struggles with being visible resonates across the participants. The study’s only male participant, Jack, explains he started losing weight after a visit to his doctor, during which he was described as overweight and at risk of heart problems. Although his body, in light of it being male, is read differently than Lea’s, Jack similarly describes a response of recognition and praise at his initial weight loss. He also describes a subsequent turning point, when the recognition transformed to concern, and his body become troubling to others. Jack explains how he finds it difficult to be recognized as having an ED as a man and having to go to therapy:

‘[Men] they have pride, they have masculinity, they have role models, which makes it more difficult for them, I think. Now I should be careful, because I haven’t met any [other men with an ED], but I think it’s a little bit easier as a woman, a girl, to say out loud that you have bulimia or anorexia.’

This section highlights the complex dynamics of bodywork and social recognition experienced by individuals with eating disorders. As seen in the narratives of Lea and Jack, the act of disciplining one’s body initially garners approval and praise, but as weight loss progresses, it shifts into a source of alarm and unease for those around them. The participants’ bodies, once celebrated for their alignment with societal ideals of control and success, become ‘troublesome’ when they deviate too far from these norms.

In both cases, the participants describe the shift from being recognized as ‘successful’ to being viewed with concern, illustrating the precarious line between the right kind of visibility and overvisibility. This ambivalence—desiring recognition yet feeling uneasy about it—reflects broader societal contradictions in how bodies, particularly those affected by eating disorders, are perceived and judged.

As the analysis moves forward, these tensions invite further exploration of how eating disorder recovery involves not only navigating one’s own relationship with the body but also managing the external gazes that shape and disrupt these experiences.

### Untroubling the Body

In the institutional space where participants convene as troublesome bodies, they encounter a group therapy that delves beyond the realm of the body—seeking to displace the locus of the problem elsewhere. The treatment paradigm is that eating disordered bodywork has arisen as a way to cope with something in life—the actual object toward which the intervention should be directed.

A significant number of participants express initial resistance to the group process. Emily, a former participant, states in the interpretation workshop after reading interview extracts from other participants:

‘Like them, I also had a fundamental fear when I started like “God, are we going to sit here and agree that this body is wrong” or something, because that’s what we all think. Somewhere, now that I think logically, when you gather people who say "we share this disorder", you might fear that it would intensify within the group. But it’s as if it doesn’t; it’s more like it dissolves. Because we are all at the same stage there, a framework is formed that one can delve into, [consisting of] other aspects, perhaps more nuances of oneself in relation to the eating disorder.’

The quote indicates that the prospect of a group process activates participants’ idea that their bodies are wrong and troubling and should be compared and measured. However, the focus they encounter in the therapy activates something entirely different: A blurring of the body and a new analysis of the issues participants are grappling with. Problem identification is not dictated by ED and the various versions of the body they feel compelled to inhabit are 'dissolved', as Emily puts it. In this space, they become individuals with diverse issues, regardless of their bodies. Discussions about the body are possible, opening up avenues for other types of conversations and new shared analyses of the problem, where the definition of the problem is not dictated by a diagnosis. Verbin (2020) suggest that one of the most dominant paradoxes in eating disorder is the paradox of power, expressed as power/weakness. Entering the group therapy activates a feeling of being out of control by being over-visibile in a way that they already struggle with and wish to break free from. Surprisingly, the participants unanimously express that meeting other troublesome bodies gives them control and power, because their bodies are not isolated, but met by a community where everyone is different/peculiar. This gives the participants an opportunity to define themselves in different ways, which is described as empowering.

As Emily states: ‘It was an environment and a community where one was not perceived as sick or eating disordered, but precisely not seen for one’s body or eating patterns or anything, but actually seen as a person who just wanted to talk.' Emily's narrative shows how the body, troubling at first sight, becomes un-troubled in the therapeutic group. At the same time, the paradoxes of mind/body explained by Verbin seem to move to the foreground. By bracketing the body—'not seen for one’s body'—it is possible to ‘just talk’ and become a person.

Another participant, Mia, describes the group process as one that uncovers the root of problems. Mia’s describes herself as someone who has struggled her whole life with what she refers to as low self-esteem. She describes a previous treatment in the Child and Adolescent Mental Health Services (CAMS) in the following way:

‘It was as if after that treatment [at CAMS] I sort of entered into such, I didn’t feel well, it was as if, um [searching for words], that [heavy breathing], what I kind of see as the *root* of all these eating issues, um, and this coping strategy like, there was a *need* for them, that I [searching for words], my sort of relationship with myself and the way I talked to myself and related to myself [heavy breathing], hasn’t been very good, um, or very constructive. And it wasn’t really strengthened throughout the treatment process, um, back then in high school. So, so because the root wasn’t really dug up, well, then it sort of continued to occupy my mind in the years after.’

In the extract, the ‘root’ of the problems is constructed as something other than the body, and as something that needs to be ‘dug up’ for the person to recover. Mia rejects the notion of the problem as bodily, and the body as purely medical. The previous treatment made her feel powerless and out of control. In the new group therapy, she herself can gain agency over what is troubling her; and where the trouble should be placed, as a way to reset the paradoxes of body/mind and control/loss of control. The notion that eating disorders are not ‘truly about food or the body’ but rather about something entirely different echoes across participants:

Anita: ‘The thing is that people, they only think it’s about wanting to be thin but it’s not that at all... if I refrained from, um, eating, then I could become completely numb because I was so exhausted and just turned into a zombie. So, I didn’t have to deal with all my, um, um, my support system, which was incredibly messy, and all my issues, which I was very upset about. [If] you are afraid of failing in other areas, you can use it [ED] as a way to well, to achieve some success elsewhere. Um, so, it’s not about getting thinner and thinner and thinner and weighing less and less and less. It’s just done as a strategy to achieve everything else. And of course, you hope to get, um, kinda, to be seen because it becomes so physical in the end. But yeah. But it’s not just about saying “oops! I think I’m too fat” (mm) “now I want to lose weight”, and then it spirals out of control. That’s not how it is (mm) for me, at least (laughs softly)’

Lea: ‘But I really think, of course, a lot has happened from then until now in relation to the eating disorder, but also mentally and also some of the things I grappled with many years before, long before food ever became an (mmm) issue, I have become wiser.’

In the group process, troubled bodies converge in the same space and lose their visibility. Norms are shifted—everyone is peculiar and troubled. The therapy emphasizes the ‘root of the problem’ as something external to the body. On one hand, the phrase ‘digging out the root of the problem’ can be interpreted as a way for individuals to define what troubles them, thereby acting with agency. On the other hand, framing what is troubling as a mental issue must be understood as a response to a broader culture that questions and disciplines the body—something the participants do not want to passively accept, but still are affected by. Their narratives reflect the paradoxes of mind/body and control/loss of control—without any certainty about how these paradoxes will resolve. Throughout the recovery process, the therapy room provides the opportunity to perform and explore these paradoxes without directly addressing them, while simultaneously granting the participants agency to take control and ownership over what is troubling them—an act closely tied to their understanding of recovery.

In the final theme, we will discuss how participants navigate and respond to concerned gazes, managing a body that is both mechanistic and sensual. During the treatment program, they are expected to consume food in regulated ways, disconnected from their sensations of hunger and desire. At the same time, they strive to develop new sensations and to cultivate a newfound, enjoyable relationship with food.

### Navigating a Mechanized Sensual Body

Eating disordered bodies can be described as hyperdisciplined. They learn to suppress and manage hunger, exhaustion, and fatigue, adhering to strict rules governing food intake; x number of calories, x number of bites of a specific food item, x minutes of exercise. The acts of eating and abstaining from food actively consume everyday life, literally eating away at the focus on everything else. The constant work makes it challenging to unwind and concentrate on other aspects of life. As Lily explains:

‘I was just really sad all the time and had a hard time relaxing and being present and then I just thought about food all the time, it just took up an incredible amount of time and energy, thoughts about food, and what I should [eat], and when I should eat, and how I should eat, and what and how much and it really just made it impossible to be relaxed about anything because it was just my focus the whole time. Then I also think I was quite hungry all the time, without really knowing it, I didn’t really have any energy and was quite tired and didn’t really want to be with anyone.’

Lily’s quote illustrates the immense demands of navigating the body through rigid, mechanical routines, which consume significant mental and physical resources. The body exists within the paradox of mind and body, and of control and power. While ‘the mind’ attempts to dominate the body, suppressing its natural needs, the body pushes back—noisy in its hunger and fatigue, resistant in its inability to relax—ultimately exerting its own control by disrupting the very discipline imposed on it.

During the treatment, the participants are confronted with requirements to change their eating patterns. Ideally, they should stop thinking about the body and body actions and set themselves ‘free.’ As Emily, a former patient from the workshop, explains: ‘I’m completely, completely recovered now, I don’t need any treatment, there’s nothing left [of the ED] at all and [I] haven’t been [in need of help] for 2 years now, I think, completely recovered, like I am not limited by anything at all’.

To achieve this sense of freedom, Emily explains, the body must be reconditioned through new eating patterns—a kind of rite of passage that can be challenging to let go of. Lily, who is nearing the end of her treatment, describes this difficulty:

‘I can feel that I still think about food like , how should I put it, like [in a] structured way. I think it might also have something to do with the treatment here. I had to change the whole way I thought about food. Um, and got into this mechanical eating, which they work a lot with out here. I think that I got a very structured idea of how you should eat. And I think it is very difficult to let go of it again. And that also means that I get enough food, but that I still, when I eat something, I pay a lot of attention to which meal it belongs to. Um, and I think that could well be a challenge I have today.’

Lily’s account shows that systematizing eating habits during recovery extends beyond the physical act of gaining weight or ensuring proper nutrition. It also reflects a desire to break free from the constant preoccupation with food and the body, what Emily calls the pursuit of 'no restrictions at all'. However, for Lily, letting go of the structured eating patterns established in treatment is difficult. This struggle likely arises from the transformation of her previous rigid control over food intake—aimed at starvation—into a new, mechanical regimen  during recovery. In treatment, she learned to nourish her body without relying on natural hunger cues, creating a paradox in recovery.

On one hand, Lily describes the need to reconnect with her inner self and desires, while on the other, she remains vigilant about adhering to the treatment’s guidelines. The paradoxical tension between 'being in touch with my body', as she calls it, and simultaneously controlling the body makes it challenging to stop overthinking food and bodily sensations.

A further complication emerges later in recovery, as the sensual experience of the body becomes increasingly troubling with weight gain. Nora explains:

‘I may have kind of overlooked the whole aspect of “okay, what happens when the body starts to gain weight?” because it feels strange, you have a stomach ache, and you also have a bloated stomach [laughter] all the time, and yes, I also remember that there was someone in the group; she also mentioned that she thought the weight was distributed a little differently. She was in a different place than I was at the time, so I didn’t dwell too much on it. But I can still recall she said that “I feel so good”, I can relate to it now when I, like, just can’t recognize my body, and then you just get to have that body and then you stand there like this, um, blobby creature and think “urgh!Now what is this?” And then you just have to go out and act normally with your, um, uncertainties, confusions - and I think that has actually been difficult.’

Nora highlights how the body undergoes changes that can be disorienting, awakening a new awareness of weight distribution. The sensual aspects of the body become troublesome, particularly when it takes on a ‘blobby’ quality. As Ahmed (2006) notes, bodies matter. Our bodies shape our interactions with the world and cannot be ignored. In this context, the sensual experience is twofold: It can be distressing when perceived as a ‘blob,’ yet, as another participant reflects, it can also be affirming when manifested in acts of purging: ‘Through vomiting, you can feel that you exist.’ The indulgent and sensory relationship with food is, paradoxically, perceived as both enticing and perilous, often demanding further discipline. As Nora explains: ‘I actually almost never eat out of desire, and if I do, I get extremely upset afterwards and then, uh, I have to compensate the next day’.

In the participants’ narratives about eating disorders, a recurring theme emerges that can be described as a form of sensory troubling. The body’s senses and signals—hunger, weight, shape—are disciplined and suppressed, yet they resurface in disruptive ways. These sensory aspects persist throughout the recovery process, manifesting as fatigue, exhaustion, vomiting, and rumbling stomachs. There is a constant concern in their narratives about navigating the body as a sensory entity, where mechanical strategies can be both beneficial and detrimental at the same time.

In the analysis above, we have indicated how the sensual aspects of the body pose a problem and become troublesome during the process of recovery. The trouble that the sensory body poses is different from the trouble that comes from external gazes and from the bracketing of the body in the space of group therapy. The participants are struggling to (re)learn a sensual relationship with their bodies, while striving to liberate themselves from compulsorily regulated thoughts about food. At the same time, they strive to maintain a systematic and mechanical eating pattern, having to eat without feeling hungry. As the body gains weight or as weight stabilizes, they must deal with what they see as a blobby and unruly body that cannot be controlled. Their bodies thus continue to be troublesome—just in new and different ways.

## Conclusion

This study highlights the complexities involved in recovering from eating disorders, particularly how the body remains a central, often paradoxical, presence throughout the process. Drawing on the narratives of participants who had participated in a group treatment informed by narrative and systemic approaches, we explored how the body becomes both an object of control and a source of disruption in recovery.

Our analysis shows that the participants have always engaged in forms of bodywork—attempts to elicit both recognition and care through their bodies. They struggle with being visible, seeking recognition as feminine or masculine subjects, while also resisting visibility to avoid concern and judgment.

Throughout their eating disorder, participants developed a mechanical relationship with their bodies, controlling sensations of hunger and disciplining themselves through exercise, or in some cases, vomiting. This mechanical control is both a means of gaining power over the body and a form of protection from the cultural and medical gazes that scrutinize their bodies. Many participants avoided socializing altogether, fearing judgment and concerned looks shaped by cultural or medical norms.

Despite their initial skepticism toward group therapy, participants ultimately experienced it as a surprising and transformative space. Initially, they feared entering a collective setting where their ‘peculiar’ bodies might be further scrutinized. However, they found that group therapy provided a rare space where they could share the experience of troublesome bodies. Paradoxically, being physically present with others in the group allowed their bodies to ‘dissolve.’ In this shared space, participants felt empowered to explore their issues without the constant preoccupation with the body. By collectively bracketing the body, they gained a sense of liberation and agency, allowing them to reclaim ownership over defining their challenges and analyzing their problems in a supportive, collective environment.

During treatment, as part of the recovery process, participants were required to engage in a predefined structured eating pattern, eating even when they weren’t hungry or didn’t desire food. Despite attempts to develop this new kind of discipline over the body in recovery, the body remains unruly—constantly asserting itself through sensations like hunger, ‘blobs’ or bloating. As participants navigate recovery, the body oscillates between being a mechanized entity that must be managed and a sensual, living organism that resists total control.

What is particularly interesting is not only how the body ‘troubles’ the participants, but also that it does not trouble in any predefined or fixed manner. While participants’ bodies continually disrupt their efforts to control them, this does not imply a lack of agency. Paradoxically—or perhaps because of this—our analysis reveals that participants experience a growing sense of agency and empowerment throughout the recovery process, particularly in group therapy. Here, they are not passive victims of their bodies but act as agentive, embodied beings, twisting and negotiating their way out of externally imposed definitions of their bodies.

As Ahmed (2006) emphasizes, bodies come into being through their interactions with others. This relational aspect may explain why group therapy is so impactful for participants. Troublesome bodies do not arise in isolation; they emerge within collective spaces where bodies interact and reflect each other. For the participants in this study, group therapy was crucial—not because it solved all their issues with EDs, but because it provided a collective space where they could engage with their troubled bodies without needing to conform to the rigid societal dichotomies they encountered elsewhere in the treatment system.

Our analysis thus suggests an expansion of Analu Verbin’s three dominant paradoxes of eating disorders (feminine/masculine, control/loss of control, body/mind) to include a fourth: The paradox of the individual/collective. In recovery, this paradox refers to the tension between personal responsibility and communal support, where participants must navigate the process of reclaiming a new type of agency over their own bodies while recognizing that this agency is shaped by and realized through shared, collective experiences. The collective context of group therapy plays a key role in this balance, offering a space where individual recovery is deeply interwoven with the presence and influence of others.

This underscores the importance of collective spaces in recovery, where participants are not merely working on their individual bodies but are shaped by, and shape, the shared experience of embodiment within the group. In these spaces, they are empowered to engage with their bodies not as fixed, isolated entities, but as dynamic, agentive bodies-in-relation. This collective aspect offers a powerful counterbalance to the isolating and individualizing pressures of ED treatment, fostering a more relational approach to recovery.

Moreover, our findings underscore the importance of recognizing that recovery does not simply involve resolving issues of control over the body. Instead, it requires an ongoing negotiation between controlling and sensing the body, between mechanical strategies and the unpredictable nature of the body’s responses. Group therapy, while helpful in creating a space where the body can ‘dissolve’, also highlights the deep-seated tensions participants face as they attempt to reconcile these two aspects of recovery.

In conclusion, this study demonstrates that recovery from an eating disorder is not a linear process but one fraught with contradictions. The body, far from being neutralized or fully disciplined, remains a complex and troublesome entity, one that requires participants to continuously navigate between control and acceptance, structure and sensation—and between the individual and collective. Understanding this dynamic is crucial for developing more nuanced therapeutic approaches that acknowledge the body’s central, and often conflicting, role in eating disorder recovery.
